# Orificial Surgery

**Published:** 1899-08

**Authors:** 


### ORIFICIAL SURGERY.

The announcement of the thirteenth annual course of instruction in Orificial Surgery by Dr. E. H. Pratt is received. According to this pamphlet the course will be held September 4th, 5th, 6th, 7th, 8th, and 9th, 1899, at the Chicago Homœopathic Medical College. Each session will last four hours. Clinics will be held with the special material supplied by the members of the class. These operations will be selected to illustrate the effect of this special kind of surgery upon cases of asthma, dyspepsia, paralysis, eczema, and other chronic diseases.

All the different operative procedures in this special surgery will be thoroughly explained and vividly illustrated.

The art of tissue reading will receive marked attention in the coming class. All sorts of capital operations will be performed and explained.

As seats will be at a premium, those who wish to attend should apply early. The classes will be divided into sub-classes of ten, which will be received into the operating room in rotation. Thus ample opportunities will be furnished to all for close observation.

The tuition will be $25.00 in advance to practitioners, for whom it is especially designed. Address Dr. E. H. Pratt, 100 State St., Chicago, Ill.

